# N‐Terminal Propeptide of Procollagen Type III (PIIINP) as a Biomarker for Liver Fibrosis Severity: A Systematic Review and Meta‐Analysis

**DOI:** 10.1155/ijpe/7410174

**Published:** 2026-11-15

**Authors:** Fatemeh Famouri, Hamideh Komeilyfard, Hossein Saneian, Peiman Nasri, Majid Khademian, Mohammad Reza Maracy

**Affiliations:** ^1^ Department of Pediatrics Imam Hossein Children Hospital Isfahan University of Medical Sciences Isfahan Iran mui.ac.ir; ^2^ Metabolic Liver Disease Research Center Imam Hossein Children Hospital Isfahan University of Medical Sciences Isfahan Iran mui.ac.ir; ^3^ Child Growth and Development Research Center Research Institute for Primordial Prevention of Non-Communicable Disease Imam Hossein Children Hospital Isfahan Iran; ^4^ Department of Epidemiology & Biostatistics School of Public Health Isfahan University of Medical Sciences Isfahan Iran mui.ac.ir

**Keywords:** biopsy, collagen Type III, liver cirrhosis, liver fibrosis, N-terminal propeptide of procollagen Type III

## Abstract

**Introduction:**

The invasive nature of liver biopsy, the gold standard for liver fibrosis assessment, underscores the critical need for robust noninvasive biomarkers. The N‐terminal propeptide of procollagen Type III (PIIINP), a direct marker of fibrogenesis, is one such candidate. Despite numerous primary studies, there has been no definitive meta‐analysis to quantitatively summarize its diagnostic performance across different fibrosis stages and etiologies. This systematic review and meta‐analysis was therefore conducted to precisely determine the pooled diagnostic accuracy of PIIINP for detecting and staging liver fibrosis and cirrhosis.

**Methods:**

We conducted a comprehensive literature search across PubMed, Scopus, Web of Science, and Embase. Eighteen studies, comprising 4639 individuals, were included for qualitative assessment and meta‐analysis. Study quality was generally high (15 studies), with two rated as fair and one as low. A total of 30 substudies were analyzed from these 18 publications, as several studies reported multiple outcome measures. The primary aim was to evaluate the diagnostic accuracy of PIIINP for detecting and staging liver fibrosis and cirrhosis.

**Results:**

The pooled diagnostic metrics for PIIINP in determining liver fibrosis severity were as follows: sensitivity, 72.29% (95% CI: 64.72–79.87); specificity, 79.34% (95% CI: 74.02–84.66); positive predictive value, 61.70% (95% CI: 49.49–73.90); negative predictive value, 81.29% (95% CI: 73.14–89.43); area under the curve, 0.75 (95% CI: 0.70–0.81); and overall accuracy, 73.29% (95% CI: 70.53–76.05). At a cutoff value of 7.34 ng/mL, PIIINP demonstrated a sensitivity of 67% (95% CI: 59–75) and a specificity of 77% (95% CI: 72–82) for differentiating advanced fibrosis or cirrhosis from milder stages.

**Conclusion:**

PIIINP demonstrates clinically useful accuracy as a noninvasive biomarker for assessing liver fibrosis severity, independent of underlying etiology. Its strong negative predictive value is particularly valuable for ruling out advanced disease. Future research should focus on validating disease‐specific cutoffs and integrating PIIINP into standardized diagnostic algorithms for targeted patient populations.

## 1. Introduction

For decades, liver biopsy has remained the gold standard for diagnosing hepatic fibrosis, yet its invasive nature and limitations including sampling variability and complications have driven the search for reliable noninvasive alternatives [1, 2].

In response, a diverse array of serological biomarkers has been developed. Established indices such as the AST‐to‐Platelet Ratio Index (APRI), the FIB‐4 score, and the Enhanced Liver Fibrosis (ELF) panel, among others, provide valuable tools for broadly distinguishing fibrotic livers. Yet, a significant limitation persists: These tests often fall short of precisely staging the degree of fibrosis, a critical factor for clinical management and prognosis [3–5]. In contrast, direct biomarkers target the core pathophysiology of fibrosis—the hepatic stellate cell–mediated deposition of extracellular matrix (ECM) components, particularly Type III collagen [6].

Among direct biomarkers, the N‐terminal propeptide of Type III procollagen (PIIINP) offers unique advantages. During collagen synthesis, PIIINP is cleaved and released into circulation, providing a direct window into active fibrogenesis [7, 8]. This mechanistic basis has established PIIINP as a promising biomarker, though its clinical application requires further validation [9].

The clinical context of PIIINP measurement warrants careful consideration. While elevated levels reflect active fibrogenesis across various etiologies including metabolic dysfunction–associated steatotic liver disease (MASLD), viral hepatitis, and alcoholic liver disease (ALD), PIIINP lacks disease specificity [10]. Furthermore, as PIIINP originates from collagen synthesis, elevated levels may occur in extrahepatic conditions involving fibroproliferative processes [11, 12].

Recent evidence underscores the systemic implications of liver fibrosis, particularly in MASLD, where endothelial dysfunction, inflammation, and fibrosis form a pathogenic triad driving both hepatic and extrahepatic complications [13–15]. The systemic inflammatory state contributes to elevated cardiovascular risk, including heart failure in younger populations, while also promoting metabolic, renal, cognitive, and musculoskeletal complications [16]. This expanded understanding highlights the importance of accurate fibrosis staging not only for hepatic prognosis but also for comprehensive metabolic and cardiovascular risk assessment.

Despite a strong pathophysiological rationale, the evidence base for PIIINP remains fragmented. Existing studies typically focus on specific patient populations and are often limited by sample size constraints [9, 17–19]. Therefore, this systematic review and meta‐analysis is aimed at synthesizing evidence from the past two decades to provide a comprehensive evaluation of PIIINP′s diagnostic and prognostic utility across the spectrum of liver fibrosis.

## 2. Methods and Materials

### 2.1. Research Strategy

This systematic review and meta‐analysis was conducted in accordance with the Preferred Reporting Items for Systematic Reviews and Meta‐Analyses (PRISMA) guidelines [20]. A comprehensive literature search was performed across four electronic databases: PubMed, Scopus, Web of Science, and Embase on January 25, 2023, covering publications from January 1, 2000, to December 30, 2022.

Studies published before the Year 2000 were excluded. This temporal cutoff was implemented to ensure the consistency and contemporary relevance of the histopathological reference standard. The period around the Year 2000 marked the widespread adoption and validation of standardized liver fibrosis staging systems (e.g., METAVIR, Ishak) as the clinical gold standard. Restricting the analysis to the post‐2000 era minimizes heterogeneity in fibrosis assessment and ensures that the diagnostic accuracy of PIIINP is evaluated against modern histological benchmarks.

The initial search yielded 226 records: 113 from PubMed, 94 from Scopus, 17 from Web of Science, and 2 from Embase. The search strategy employed consistent concepts across databases, combining terms related to the biomarker (“N‐terminal propeptide of procollagen type III,” “Pro‐C3,” and “PIIINP”) with terms for the target condition (“liver fibrosis,” “hepatic fibrosis,” “liver cirrhosis,” and “hepatic cirrhosis”).

Specific search syntax for each database was as follows:

1. PubMed: (“N‐terminal propeptide of procollagen type III”[All Fields] OR “Pro‐C3”[All Fields] OR “PIIINP”[All Fields]) AND (“liver fibrosis”[All Fields] OR “hepatic fibrosis”[All Fields]) AND (“liver cirrhosis”[All Fields] OR “hepatic cirrhosis”[All Fields])
2. Scopus: TITLE‐ABS‐KEY((“N‐terminal propeptide of procollagen type III” OR “Pro‐C3” OR “PIIINP”) AND (“liver fibrosis” OR “hepatic fibrosis”) AND (“liver cirrhosis” OR “hepatic cirrhosis”))
3. Web of Science: TS = ((“N‐terminal propeptide of procollagen type III” OR “Pro‐C3” OR “PIIINP”) AND (“liver fibrosis” OR “hepatic fibrosis”) AND (“liver cirrhosis” OR “hepatic cirrhosis”))
4. Embase: (“n‐terminal propeptide of procollagen type iii”: ti, ab, kw OR “pro‐c3”: ti, ab, kw OR “piiinp”: ti, ab, kw) AND (“liver fibrosis”: ti, ab, kw OR “hepatic fibrosis”: ti, ab, kw) AND (“liver cirrhosis”: ti, ab, kw OR “hepatic cirrhosis”: ti, ab, kw)

### 2.2. Inclusion and Exclusion Criteria

Studies were selected based on the following inclusion criteria:

1. Investigation of adult patients with chronic liver disease, with liver fibrosis or cirrhosis confirmed by histology (biopsy) or transient elastography;
2. Measurement of serum PIIINP levels;
3. Reporting of sufficient data to calculate diagnostic accuracy measures (e.g., sensitivity, specificity, or data to construct 2 × 2 contingency tables).

The exclusion criteria were as follows:

1. Review articles, meta‐analyses, editorials, or conference abstracts;
2. Non‐English publications;
3. Studies published before January 1, 2000;
4. Studies focusing on nonhepatic conditions (e.g., cardiovascular diseases, rheumatological disorders, or other extrahepatic fibrotic conditions);
5. Studies with overlapping populations or duplicate datasets.

### 2.3. Study Selection

The study selection process was conducted in accordance with PRISMA guidelines. Two reviewers independently screened the retrieved records based on title and abstract. Any discrepancies between the reviewers were resolved through discussion or, when necessary, by consultation with a third senior reviewer to reach consensus.

Following the initial screening, the full texts of potentially eligible articles were obtained and thoroughly assessed against the predefined eligibility criteria. Throughout this process, duplicate publications were identified and removed using a systematic approach to ensure each unique study was represented only once in the analysis. Finally, all studies that met all inclusion criteria without meeting any exclusion criteria were included in the qualitative synthesis (Table 1).

**Table 1:** Key components of the review questions.

Parameters	Criteria
Population	Patients with hepatic fibrosis or cirrhosis
Exposure	N‐procollagen end Peptide III
Comparator	The sensitivity, specificity, positive predictive value, negative predictive value, accuracy, area under the curve of N‐procollagen end Peptide III
Outcome	The severity of hepatic fibrosis/cirrhosis

### 2.4. Data Extraction

Data were independently extracted from the included studies by two authors using a standardized data collection form. The extracted information included the following:

• First author and publication year
• Study population characteristics
• Methods used for liver fibrosis/cirrhosis assessment
• Study objectives and endpoints
• Diagnostic accuracy parameters (sensitivity, specificity, and AUC values)
• PIIINP assay methodology and cutoff values
• Reference standard details (biopsy technique or elastography parameters)

Any discrepancies in data extraction were resolved through consensus discussions between the two authors, with involvement of a third author when necessary to ensure accuracy and consistency.

### 2.5. Statistical Analysis

A meta‐analysis of diagnostic accuracy was performed using a bivariate random effects model, which accounts for both within‐study and between‐study variabilities while incorporating the potential correlation between sensitivity and specificity. This approach is recommended for synthesizing diagnostic test accuracy data.

Heterogeneity was quantitatively assessed using the *I* ^2^ statistic, with values exceeding 50% considered indicative of substantial heterogeneity. Additional measures included Cochran′s *Q* test and the *H* ^2^ statistic, with a *p* value < 0.05 for the *Q* test suggesting significant heterogeneity. To visually identify outliers contributing to heterogeneity, a Galbraith plot was generated, displaying standardized effect sizes against their precision (1/standard error).

We conducted prespecified subgroup analyses according to fibrosis severity stages: mild fibrosis, significant fibrosis, advanced fibrosis, and cirrhosis. Sensitivity analyses were performed to evaluate the robustness of the pooled estimates. Publication bias was assessed through visual inspection of funnel plots and statistical evaluation using Egger′s and Begg′s tests. Additionally, a nonparametric trim‐and‐fill analysis was implemented to estimate the potential impact of missing studies. All analyses were conducted using Stata software Version 17.

## 3. Results

### 3.1. Study Selection

The literature search strategy yielded 226 records that 99 have remained after duplicates removal. After initial screening according to the titles and abstracts, 72 full‐text articles were saved and assessed. Ultimately, 18 studies were included in the qualitative assessment and meta‐analysis study as shown in the PRISMA flow diagram in Figure 1 and Table 2.

**Figure 1:**
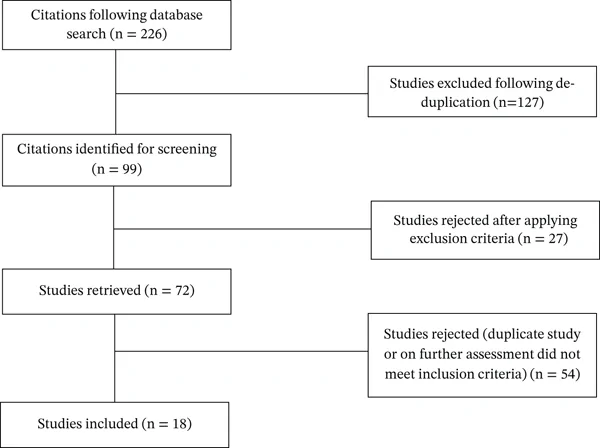
Flowchart illustrating the summary of literature search results.

**Table 2:** The detailed extracted data of the included studies.

Number	Author	Country	Sample size	Type of study	Gold standard	Diagnostic criteria: Grading	Laboratory assessment	Age	Assessment	Disease
1	Hansen et al. [25]	Denmark	270	Cross‐sectional	Biopsy	Ishak score: ≥ 3 significant fibrosis; ≥ 5 cirrhosis	ELISA	43	Fibrosis	HCV
2	Martyn et al. [29]	United Kingdom	27	Pre‐poststudy	Biopsy	Ishak score: < 3 mild fibrosis, ≥ 3 significant fibrosis	Radioimmunoassay	56 ± 2.7	Fibrosis	Methotrexate‐induced hepatotoxicity
3	Haung et al. [20]	China	1565	Cross‐sectional	Biopsy	METAVIR: 2 significant fibrosis; 3 advanced fibrosis; 4 cirrhosis	Electrophoresis	—	Fibrosis	HBV
4	Dold et al. [33]	Spain	116	Cross‐sectional	Transient elastography	METAVIR: ≥ 2 significant fibrosis	ELISA	47.3	Fibrosis	HIV
5	Omran et al. [16]	Egypt	181	Cross‐sectional	Transient elastography	METAVIR: ≥ 3 advanced fibrosis	ELISA	51.6 ± 9.9	Fibrosis	HCV
6	Nielsen et al. [26]	Spain	432	Pre‐poststudy	Biopsy	METAVIR: =2 significant fibrosis, ≥ 3 advanced fibrosis	ELISA	43.5	Fibrosis	HCV
7	Nassef et al. [27]	Egypt	60	Case–control	Biopsy	METAVIR: ≥ 2 significant fibrosis	ELISA	—	Fibrosis	HCV
8	Cheong et al. [21]	Korea	367	Cross‐sectional	Biopsy	METAVIR: 4 cirrhosis	ELISA	41.84 ± 12.61	Cirrhosis	HBV and HCV
9	Valva et al. [28]	Argentina	22	Cross‐sectional	Biopsy	METAVIR: =2 significant fibrosis, ≥ 3 advanced fibrosis	ELISA	Median: 8 (range: 1–17)	Fibrosis	HCV
10	Dong et al. [22]	China	70	Cross‐sectional	Biopsy	Modified Scheuer scoring system: ≥ 2 significant fibrosis	Electrochemiluminescence immunoassay	38.5	Fibrosis	HBV
11	Chen et al. [23]	China	108	Case–control	Biopsy	Modified Scheuer scoring system: < 2 mild fibrosis	ELISA	34.23 ± 9.84	Fibrosis	HBV
12	Zheng et al. [24]	China	114	Cross‐sectional	Biopsy	Modified Scheuer scoring system: < 2 mild fibrosis	Radioimmunoassay	—	Fibrosis	HBV
13	Gawrieh et al. [30]	India	166	Randomized clinical trial	Biopsy	NASH Clinical Research Network: ≥ 2 significant fibrosis	—	13.1 ± 2.4	Fibrosis	NAFLD
14	Bril et al. [31]	United States	162	Case–control	Biopsy	NASH Clinical Research Network: ≥ 3 advanced fibrosis	ELISA	57.71 ± 8.33	Fibrosis	NAFLD
15	Mosca et al. [7]	Italy	204	Cross‐sectional	Biopsy	NASH Clinical Research Network: =2 significant fibrosis; ≥ 3 advanced fibrosis	ELISA	—	Fibrosis	NAFLD
16	Bril et al. [32]	United States	191	Cohort	Biopsy	NASH Clinical Research Network: =2 significant fibrosis; ≥ 3 advanced fibrosis	ELISA	59 ± 8	Fibrosis	NAFLD
17	Bel Lassen et al. [17]	France	158	Pre‐poststudy	Biopsy	Steatosis Activity and Fibrosis: ≥ 3 advanced fibrosis	ELISA	42 ± 12	Fibrosis	NAFLD
18	Madsen et al. [18]	Denmark	426	Cross‐sectional	Biopsy	The Kleiner fibrosis stage and nonalcoholic fatty liver disease activity score: ≥ 3 advanced fibrosis	ELISA	56.5 ± 10.5	Fibrosis	ALD

Abbreviations: ALD, alcohol‐induced liver disease; ELISA, enzyme‐linked immunosorbent assay; HBV, Hepatitis B virus; HCV, Hepatitis C virus; HIV, human immunodeficiency virus; NAFLD, nonalcoholic fatty liver disease; NASH, nonalcoholic steatohepatitis.

In the meta‐analysis, separate forest plots were presented for outcomes regarding hepatic fibrosis/cirrhosis based on the cut points of PIIINP to determine the severity of hepatic fibrosis/cirrhosis.

Results of quality assessment of included studies were depicted in Table 3. As it is shown, the majority of included studies had high quality (15 studies), and two studies had fair quality, and only one included study in the systematic review had low quality.

**Table 3:** The quality of studies.

Number	Author		1	2	3	4	5	6	7	8	9	10	11	12	13	14	Quality
1	Hansen et al. [25]	Cross‐sectional	✓	✓	✓	✓	✓	✓	✓	✓	✓	✓	✓	×	✓	✓	Good
2	Martyn et al. [29]	Pre‐poststudy	✓	✓	✓	✓	✓	✓	✓	✓	✓	✓	✓	✓	—	—	Good
3	Haung et al. [20]	Cross‐sectional	✓	✓	✓	✓	✓	✓	✓	✓	✓	✓	✓	✓	✓	✓	Good
4	Dold et al. [33]	Cross‐sectional	✓	✓	✓	✓	✓	✓	✓	✓	✓	NA	✓	×	✓	✓	Good
5	Omran et al. [16]	Cross‐sectional	✓	✓	✓	✓	✓	✓	✓	✓	✓	NA	✓	×	✓	✓	Good
6	Nielsen et al. [26]	Pre‐poststudy	✓	✓	✓	✓	✓	✓	✓	✓	✓	✓	✓	✓	—	—	Good
7	Nassef et al. [27]	Case–control	✓	✓	✓	✓	✓	✓	✓	✓	✓	✓	✓	✓	—	—	Good
8	Cheong et al. [21]	Cross‐sectional	✓	✓	✓	✓	✓	✓	✓	✓	✓	NA	✓	×	✓	✓	Good
9	Valva et al. [28]	Cross‐sectional	✓	✓	✓	✓	✓	✓	✓	✓	✓	NA	✓	✓	✓	✓	Good
10	Dong et al. [22]	Cross‐sectional	✓	✓	✓	✓	✓	✓	✓	✓	✓	NA	✓	✓	✓	✓	Good
11	Chen et al. [23]	Case–control	✓	✓	✓	✓	✓	×	✓	✓	NA	✓	✓	NA	—	—	Fair
12	Zheng et al. [24]	Cross‐sectional	✓	✓	×	✓	✓	NA	✓	NA	✓	NA	✓	✓	✓	✓	Good
13	Gawrieh et al. [30]	Randomized clinical trial	✓	✓	✓	✓	✓	✓	✓	✓	✓	✓	✓	✓	✓	✓	Good
14	Bril et al. [31]	Case–control	✓	✓	✓	✓	✓	✓	✓	✓	✓	✓	✓	✓	—	—	Good
15	Mosca et al. [7]	Cross‐sectional	✓	✓	✓	✓	×	✓	✓	✓	✓	NA	✓	✓	✓	✓	Good
16	Bril et al. [32]	Cohort	✓	✓	✓	×	×	✓	✓	×	×	×	✓	✓	✓	×	Poor
17	Bel Lassen et al. [17]	Pre‐poststudy	✓	✓	✓	✓	✓	✓	✓	✓	✓	✓	✓	✓	—	—	Good
18	Madsen et al. [18]	Cross‐sectional	✓	✓	✓	✓	✓	✓	✓	✓	✓	✓	✓	✓	✓	✓	Good

Abbreviation: NA, not applicable.

The sample size of the study includes 4639 individuals. From all 18 studies, we extracted 30 substudies because each study may include more than one outcome measurement (Table 2).

The main reported characteristics and data in the included articles are demonstrated in Table 2. All the included studies have been conducted on human samples. The studies included 1 randomized clinical trial (5.5%), 3 pre–postinterventional studies (16.6%), 10 cross‐sectional studies (55.5%), 1 cohort study (5.5%), and 3 case–control studies (16.6%) (Table 2).

Liver fibrosis/cirrhosis was evaluated on populations including patients with HBV [21–25], HCV [17, 22, 26–29], methotrexate‐induced hepatotoxicity [30], nonalcoholic fatty liver disease (NAFLD) [8, 18, 31–33], human immunodeficiency virus (HIV) [34], and ALD [19]. In order to determine the severity of the liver fibrosis/cirrhosis, the majority of the studies applied liver biopsy [8, 18, 19, 21–29, 31–33] as the gold standard, while transient elastography was utilized by Dold et al. [34] and Omran et al. [17]. The instruments by which hepatic fibrosis/cirrhosis grading was interpreted included Ishak score [26, 30], METAVIR [17, 21, 22, 27–29, 34], modified Scheuer scoring system [23–25], NASH Clinical Research Network [8, 31–33], Steatosis Activity and Fibrosis [18], and the Kleiner fibrosis stage and NAFLD activity score [19]. Furthermore, we have determined the definitions applied by each of the studies as the grade of fibrosis in Table 2 (diagnostic criteria; grading column) to show the diversities used by the authors to determine the level of fibrosis. Besides, the laboratory method by which PIIINP was measured included enzyme‐linked immunosorbent assay (ELISA) [8, 17–19, 22, 24, 26–29, 32–34], radioimmunoassay [25, 30], electrophoresis [21], and electrochemiluminescence immunoassay [23] (Table 2).

### 3.2. Study Quality

The National Heart, Lung, and Blood Institute (NIH) Study Quality Assessment Tool was applied to determine the quality of the studies which revealed good, fair, and poor quality for 59, 9, and 4 included studies shown in Table 3.

### 3.3. Outcomes

*Sensitivity*. The sensitivity of PIIINP to diagnose mild fibrosis was 66.70% (95% CI: 50.70–82.70) that was evaluated by Chen et al. [24] only. Significant fibrosis was evaluated in eight studies revealing a sensitivity of 61.91% (95% CI: 49.93–73.90). Advanced fibrosis was evaluated in seven studies which revealed a specificity of 84.20% (95% CI: 76.61–91.79). Hansen and colleagues [26] evaluated the value of this biomarker to diagnose cirrhosis representing a specificity of 65.70% (95% CI: 50–81.40). The overall measured specificity of PIIINP to determine the severity of liver fibrosis accounted for 72.29% (95% CI: 64.72–79.87). Generally, the gathered data were heterogeneous considering the *I* ^2^ of 93.34%. The assessment of group differences revealed significant statistical differences (*p* value = 0.01) (Figure 2a). Figure 2a depicts the dispersion of the studies in the assessment of PIIINP sensitivity showing that none of the studies was out of 95% confidence interval limits. The small‐study effect was assessed using Begg and Egger tests. A beta value of −3.29 and a corresponding *p* value of < 0.001 were calculated in the Egger test. Kendall′s *τ* was 23, with a two‐tailed *p* value of 0.36 in Begg test. And finally, the nonparametric analysis of trim and fill was done representing no missed study affecting specificity measurements.

Figure 2The analysis of PIIINP use to distinguish various degrees of liver fibrosis (detailed information is presented in Table 2; column diagnostic criteria, grading): (a) sensitivity, (b) specificity, (c) positive predictive value, (d) negative predictive value, (e) area under the curve, and (f) accuracy.

**Figure:**
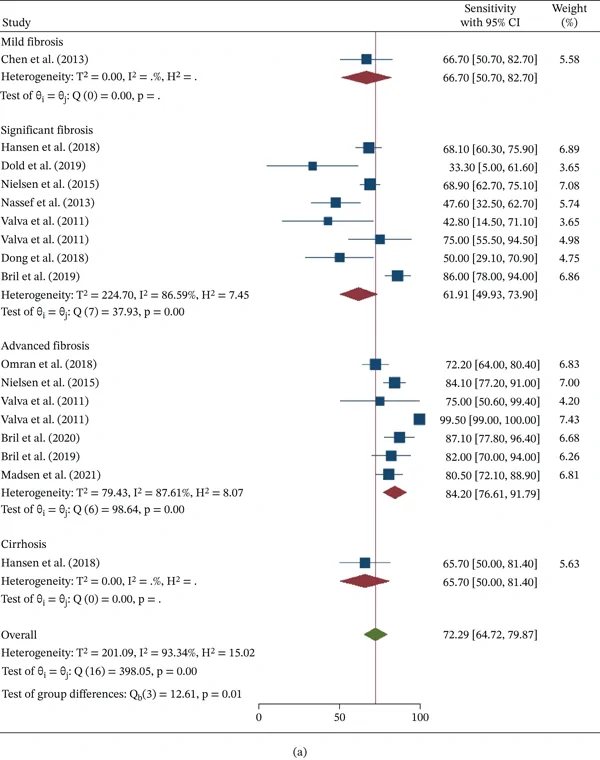

**Figure:**
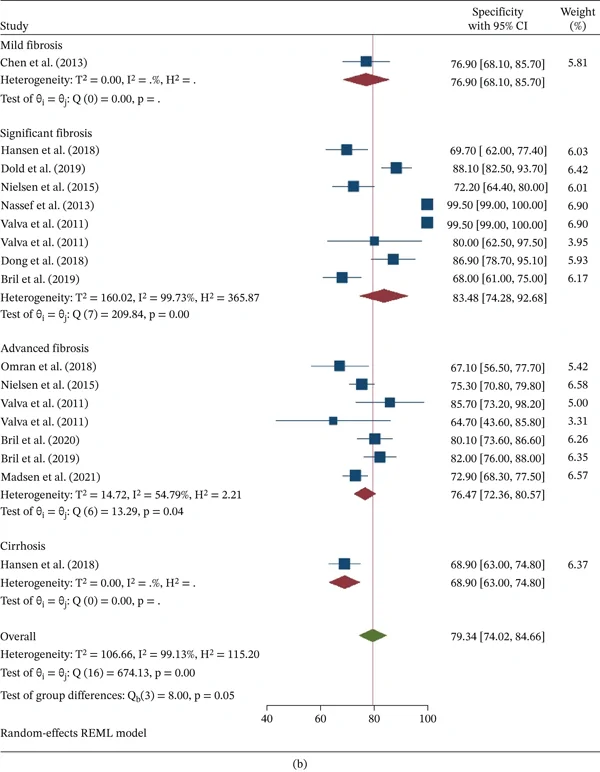

**Figure:**
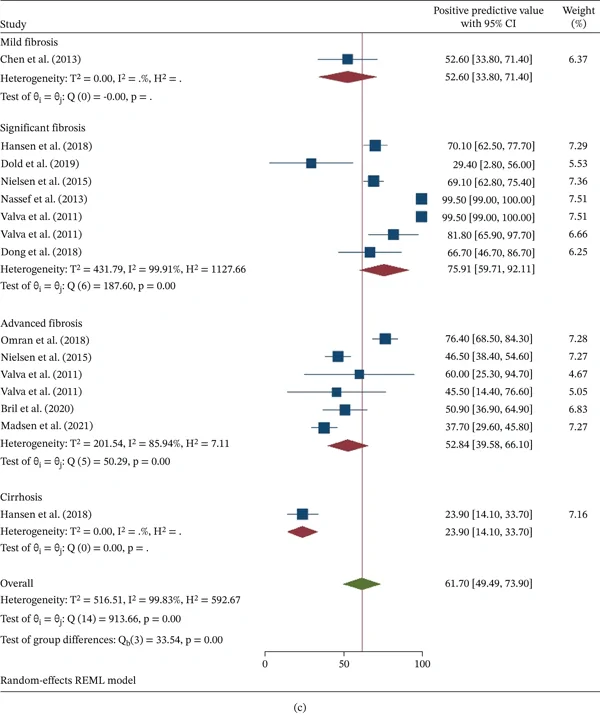

**Figure:**
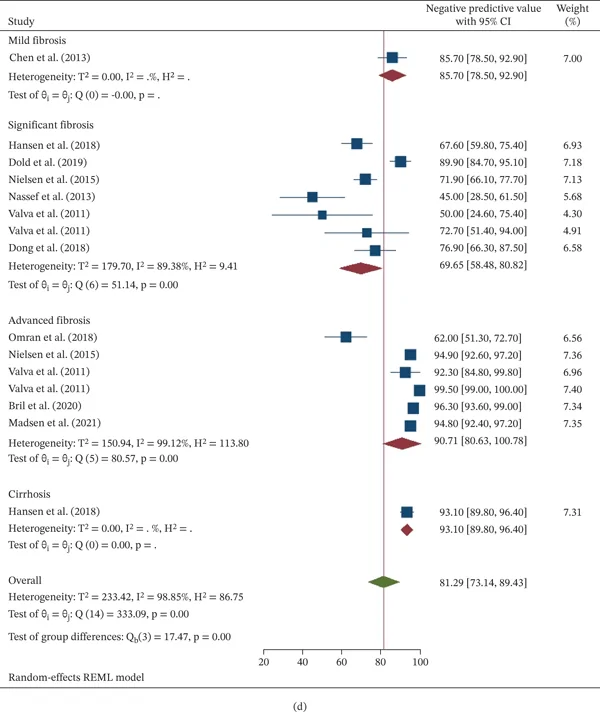

**Figure:**
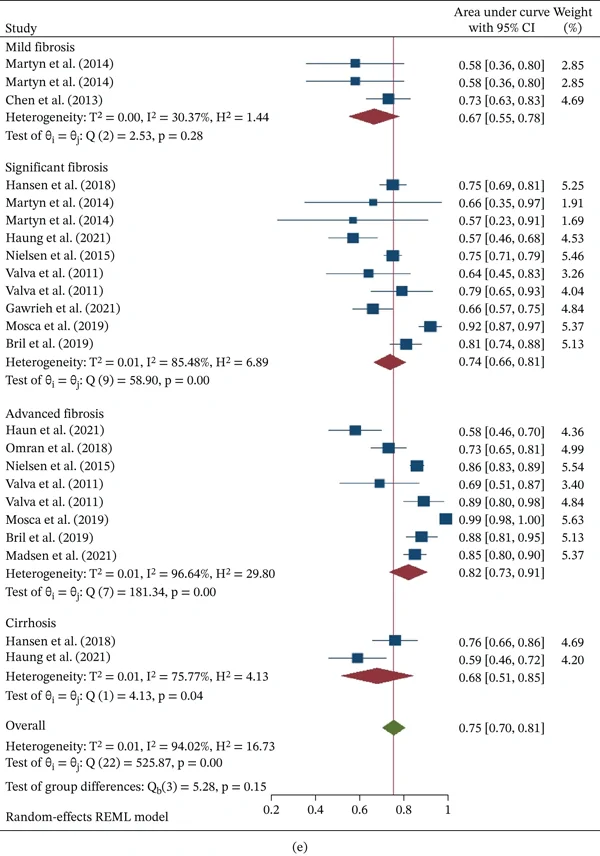

**Figure:**
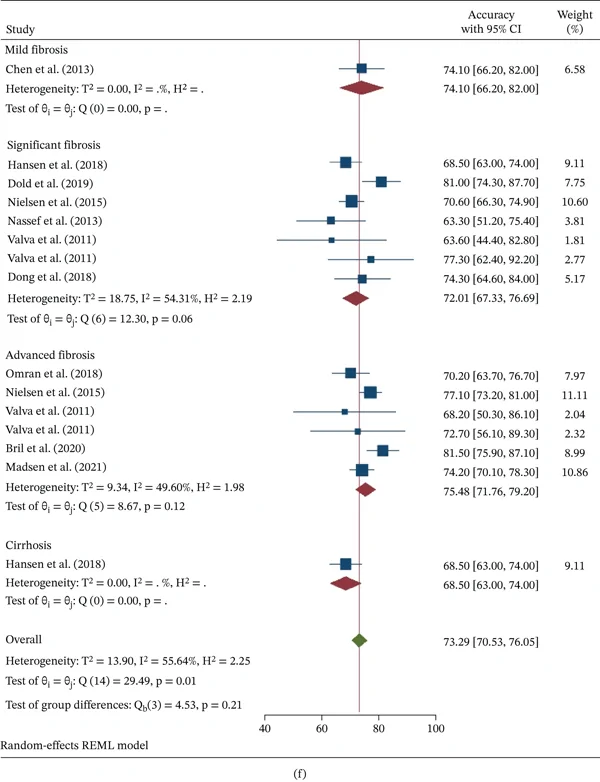

*Specificity*. The specificity of PIIINP to diagnose mild fibrosis was 76.90% (95% CI: 68.10–85.70) that was evaluated by Chen et al. [24] only. Eight studies investigated the specificity of PIIINP in diagnosing significant fibrosis that revealed 83.48% (95% CI: 74.28–92.68). Advanced fibrosis was evaluated in seven studies which revealed the specificity of 76.47% (95% CI: 72.36–80.57). Cirrhosis was evaluated in the study of Hansen and colleagues [26] representing a specificity of 68.90 (95% CI: 63–74.80). The overall measured specificity of PIIINP to determine the severity of liver fibrosis accounted for 79.34% (95% CI: 74.02–84.66). Generally, the gathered data were heterogeneous considering the *I* ^2^ of 99.13%. The assessment of group differences revealed no statistical differences (*p* value = 0.05) (Figure 2b). Figure 2b depicts the dispersion of the studies in the assessment of PIIINP specificity showing that none of the studies was out of 95% confidence interval limits. The small‐study effect was assessed using Begg and Egger tests. A beta value of −2.47 and a corresponding *p* value of 0.0191 were calculated in the Egger test. Kendall′s *τ* was 32, with a two‐tailed *p* value of 0.32 in Begg test. And finally, the nonparametric analysis of trim and fill was done representing no missed study affecting specificity measurements.

*Positive predictive value (PPV)*. The PPV of PIIINP to diagnose mild fibrosis was by Chen et al. [24] only revealing a value of 52.60% (95% CI: 33.80–71.40). Significant fibrosis was evaluated in seven studies reporting a PPV of 75.91% (95% CI: 59.71–92.11). Advanced fibrosis was evaluated in six studies which revealed a specificity of 52.84% (95% CI: 39.58–66.10). Hansen and colleagues [26] evaluated the value of this biomarker to diagnose cirrhosis with a low PPV of 23.90% (95% CI: 14.10–33.70). The overall measured PPV of PIIINP to determine the severity of liver fibrosis accounted for 61.70% (95% CI: 49.49–73.90). Generally, the gathered data were heterogeneous considering the *I* ^2^ of 99.83%. The assessment of group differences revealed significant statistical differences (*p* value < 0.001) (Figure 2c). Figure 2c depicts the dispersion of the studies in the assessment of PIIINP PPV showing that none of the studies was out of 95% confidence interval limits. The small‐study effect was assessed using Begg and Egger tests. A beta value of −2.22 and a corresponding *p* value of 0.06 were calculated in the Egger test. Kendall′s *τ* was 11, with a two‐tailed *p* value of 0.61 in Begg test. And finally, the nonparametric analysis of trim and fill was done representing no missed study affecting specificity measurements.

*Negative predictive value (NPV)*. The NPV of PIIINP to diagnose mild fibrosis by Chen et al. [24] only revealed a value of 85.70% (95% CI: 78.50–92.20). Significant fibrosis was evaluated in seven studies reporting an NPV of 69.65% (95% CI: 58.48–80.82). Advanced fibrosis was evaluated in six studies which revealed a specificity of 90.71% (95% CI: 80.63–100.78). Hansen and colleagues [26] evaluated the value of this biomarker to diagnose cirrhosis with an NPV of 93.10% (95% CI: 89.80–96.40). The overall measured NPV of PIIINP to determine the severity of liver fibrosis accounted for 81.29% (95% CI: 73.14–89.43). Generally, the gathered data were heterogeneous considering the *I* ^2^ of 98.85%. The assessment of group differences revealed significant statistical differences (*p* value < 0.001) (Figure 2d). Figure 2d depicts the dispersion of the studies in the assessment of PIIINP NPV showing that one of the studies was out of 95% confidence interval limits. The small‐study effect was assessed using Begg and Egger tests. A beta value of −4.43 and a corresponding *p* value of < 0.001 were calculated in the Egger test. Kendall′s *τ* was −39, with a two‐tailed *p* value of 0.06 in Begg test. And finally, the nonparametric analysis of trim and fill was done representing no missed study affecting specificity measurements.

*Area under the curve*. The information regarding AUC is demonstrated in Figure 2e. The overall measured AUC of PIIINP to determine the severity of liver fibrosis accounted for 75% (95% CI: 70–81). Generally, the gathered data were heterogeneous considering the *I* ^2^ of 94.02%. The assessment of group differences revealed no statistical differences (*p* value < 0.001). Figure 2e depicts the dispersion of the studies in the assessment of PIIINP AUC. The small‐study effect was assessed using Begg and Egger tests. A beta value of −2.68 and a corresponding *p* value of < 0.001 were calculated in the Egger test. Kendall′s *τ* was 28, with a two‐tailed *p* value of 0.47 in Begg test. And finally, the nonparametric analysis of trim and fill was done representing no missed study affecting specificity measurements.

*Accuracy*. The information regarding accuracy is demonstrated in Figure 2f. The overall measured AUC of PIIINP to determine the severity of liver fibrosis accounted for 73.29% (95% CI: 70.53–76.05). Generally, the gathered data were heterogeneous considering the *I* ^2^ of 55.64%. The assessment of group differences revealed no statistical differences (*p* value = 0.21). Figure 2f depicts the dispersion of the studies in the assessment of PIIINP AUC. The small‐study effect was assessed using Begg and Egger tests. A beta value of −0.70 and a corresponding *p* value of 0.35 were calculated in the Egger test. Kendall′s *τ* was −8, with a two‐tailed *p* value of 0.72 in Begg test. And finally, the nonparametric analysis of trim and fill was done representing no missed study affecting specificity measurements.

*Overall assessment*. Figure 3 shows that PIIINP at a cut point of 7.34 ng/mL had a sensitivity and specificity of 67% (95% CI: 59–75) and 77% (95% CI: 72–82) to differentiate those with advanced fibrosis/cirrhosis from the others.

**Figure 3:**
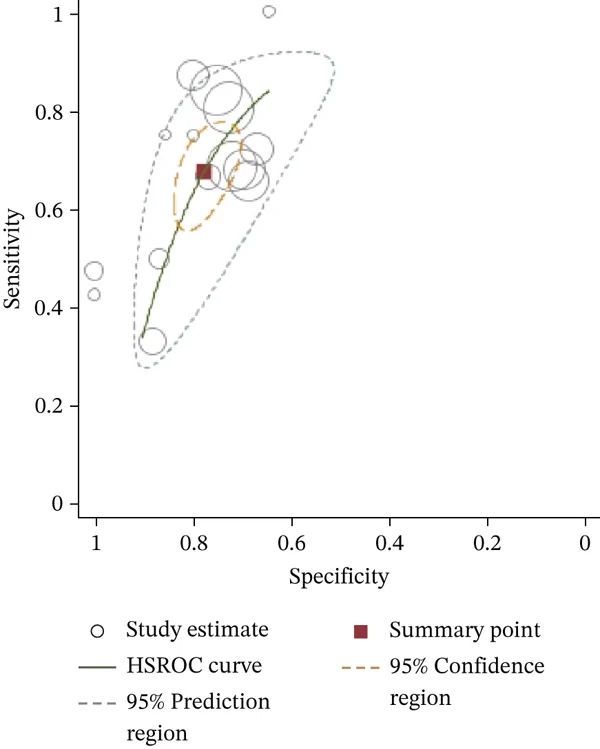
The ROC curve of PIIINP to differentiate significant fibrosis from less advanced fibrosis and more.

## 4. Discussion

This systematic review and meta‐analysis demonstrates that serum PIIINP is a valuable biomarker for staging liver fibrosis, with particularly robust performance in identifying advanced fibrosis and cirrhosis across various etiologies. The key finding of a pooled specificity of 77% and a notably high NPV of 81.29% suggests that PIIINP is most clinically useful for its ability to rule out advanced disease, which could help reduce unnecessary liver biopsies in a significant proportion of patients.

Our findings on the strong performance of PIIINP for advanced fibrosis are consistent with its well‐established biological role as a direct remnant of active collagen Type III synthesis during fibrogenesis [35]. However, the considerable heterogeneity in its diagnostic accuracy for significant fibrosis underscores a critical challenge. The wide range of reported sensitivities (33%–86%) and specificities (68%–99.5%) is not random but can be largely attributed to specific methodological variations across the included studies.

We critically evaluated this and conclude that the variability in diagnostic criteria (e.g., METAVIR F2 vs. Ishak ≥ 3 for “significant fibrosis”) is a major contributor that likely skews the pooled results. Studies using more inclusive definitions will inherently show higher sensitivity but lower specificity. This variability means our pooled estimates represent an average across different clinical definitions, and applying a single PIIINP cutoff in practice requires careful calibration to the local histopathological standard used. Furthermore, the stark contrast in performance between studies—from a sensitivity of 33% [34] to 86% [33]—can be attributed to this diagnostic heterogeneity, combined with differences in patient populations (e.g., NAFLD vs. HIV) and reference standards (biopsy vs. elastography). The observation by Valva et al. [29] of age‐dependent PIIINP levels further complicates cross‐study comparisons and highlights an often‐overlooked confounding variable.

Another important finding was the relative scarcity of data on early, mild fibrosis stages, with only one study by Chen et al. [24] reporting on this. The moderate sensitivity (61.91%) in that study suggests that PIIINP may be less effective in detecting the initial phases of fibrogenesis.

The summarized accuracy metrics provide an evidence‐based foundation for the use of PIIINP in clinical practice. Its high NPV could make it a useful “rule‐out” tool in sequential diagnostic algorithms. For researchers, our work underscores the urgent need for standardization. Future studies should focus on establishing etiology‐specific and assay‐specific cutoff values and validating PIIINP in direct comparison to other noninvasive tests like FIB‐4 or ELF score within well‐defined prospective cohorts.

The robust performance of PIIINP for advanced fibrosis is mechanistically grounded in its role as a direct remnant of active collagen Type III synthesis, a cornerstone of progressive fibrogenesis [35]. In detail, Type III collagen plays a critical role in the restructuring of the ECM, and its excessive deposition contributes to the development of fibrotic scars in various organs, including the liver and kidneys. During the synthesis of this collagen, the precursor molecule procollagen Type III is processed by enzymes. A byproduct of this cleavage is the PIIINP, which is released into the bloodstream. The concentration of PIIINP in the circulation directly reflects the rate of Type III collagen production, as each molecule of deposited collagen releases one molecule of this peptide. Consequently, serum PIIINP has been validated as a reliable indicator of fibrotic activity in liver diseases. Its circulating levels show a steady increase as hepatic fibrosis advances, demonstrating a strong positive correlation with the disease′s severity [36, 37].

This positions PIIINP distinctly within the landscape of noninvasive liver fibrosis assessment. Unlike indirect serum biomarkers (e.g., APRI and FIB‐4) that primarily reflect alterations in liver function, inflammation, and portal hypertension, PIIINP is a direct biomarker derived from the fibrogenic process itself. Similarly, while imaging‐based techniques like elastography (e.g., FibroScan and ARFI) are highly effective at measuring liver stiffness as a surrogate of accumulated scar tissue and advanced architectural distortion, PIIINP may be more sensitive to the dynamic, active phase of collagen deposition. Consequently, PIIINP′s unique value likely lies in its ability to detect active fibrogenesis, potentially identifying patients at risk of progression before irreversible structural changes are firmly established by stiffness‐based modalities [38, 39]. From a clinical perspective, the high NPV of PIIINP suggests its most immediate utility is in a sequential diagnostic algorithm. A negative PIIINP result could reliably prevent unnecessary liver biopsies in a significant number of patients with low to moderate pretest probability of advanced disease, thereby reducing patient risk and healthcare costs.

Nevertheless, it is crucial to note that our conclusions regarding the diagnostic performance of PIIINP for mild fibrosis and cirrhosis are based on a single study for each stage, which severely limits the robustness and generalizability of these findings.

To the best of our knowledge, this is the first meta‐analysis to systematically investigate PIIINP across all fibrosis severity stages and multiple liver disease etiologies, providing a comprehensive, head‐to‐head comparison of its diagnostic profile. To build on this work, future studies must prioritize standardization. This includes validating PIIINP against a single, consensus histopathological system (e.g., METAVIR) within large, prospective, and etiology‐specific cohorts encompassing the full spectrum of fibrosis, from mild stages to established cirrhosis. Furthermore, research should focus on developing integrated models that combine PIIINP with other biomarkers or clinical parameters to create a “fibrosis signature” that could surpass the diagnostic accuracy of any single test.

Another issue regarding the use of noninvasive tests to diagnose liver fibrosis is combining the tests. The accurate staging of liver fibrosis remains a cornerstone of managing chronic liver diseases, driving the need for reliable and accessible noninvasive tests. While single biomarkers like PIIINP provide valuable insights, their ultimate clinical utility is often determined by how they compare to and integrate with established panels. As recently highlighted in reviews on MASLD, the diagnostic landscape is increasingly dominated by biomarker panels like the FIB‐4 index and the ELF test, which combine multiple parameters to improve accuracy [39]. Our findings, which confirm the strong diagnostic performance of PIIINP for advanced fibrosis—particularly its high NPV—invite a critical comparison with these standard noninvasive tests. The FIB‐4 index, leveraging routine clinical parameters (age, ALT, AST, and platelets), is widely endorsed as an initial screening tool due to its high accessibility and utility for ruling out advanced disease [38]. In contrast, PIIINP offers a distinct advantage as a direct marker of fibrogenic activity, potentially capturing dynamic disease progression earlier than indirect markers that reflect hepatic inflammation and function. The ELF test, which itself incorporates PIIINP along with hyaluronic acid and TIMP‐1, represents a more advanced and validated panel [39]. Our results suggest that standalone PIIINP holds significant diagnostic power, supporting its role as the key driver of the ELF score′s performance. This positions PIIINP not necessarily as a replacement for these tests, but as a complementary tool. For instance, in a sequential algorithm, a high PIIINP level in a patient with an indeterminate FIB‐4 score could significantly increase the certainty of significant fibrosis, prompting further action or referral.

We acknowledge several limitations. First, as discussed, the heterogeneity in diagnostic criteria is a fundamental limitation that precludes the establishment of a universal PIIINP cutoff value and urges caution in interpreting our pooled estimates. Second, the paucity of data for specific fibrosis stages, particularly mild fibrosis and cirrhosis, where our analysis relied on only one study each, prevents firm conclusions. Third, the inherent inaccuracies of liver biopsy (sampling error and interpreter variability) and transient elastography (interobserver bias and failure in obesity) in the primary studies introduce an unavoidable layer of reference standard bias. Fourth, the small sample size of specific etiological subgroups, particularly the NAFLD/overweight population, limits the robustness of our conclusions for this increasingly prevalent patient group and may underpower the analysis to detect true effect sizes specific to metabolic liver disease. Finally, the use of ROC‐derived optimal cutoffs in primary studies often leads to overoptimistic performance estimates that may not generalize to broader clinical settings. Furthermore, although we assessed the methodological quality of the included studies, we did not conduct a formal analysis to examine the impact of study quality on the pooled estimates. This limitation should be considered when interpreting our results, and future meta‐analyses would benefit from such an exploration to better understand potential sources of bias and heterogeneity.

## 5. Conclusion

In conclusion, this meta‐analysis establishes PIIINP as a clinically valuable biomarker with a distinct niche: Its high NPV makes it an excellent tool for ruling out advanced liver fibrosis, potentially sparing a large number of patients from invasive biopsies. While heterogeneity in diagnostic criteria remains a challenge, PIIINP′s direct reflection of fibrogenic activity offers a unique advantage over indirect markers. Future research must focus on standardizing its use and integrating it into multiparameter diagnostic algorithms to fully realize its potential in personalized patient management.

## Author Contributions

F.F. contributed to the literature search, clinical studies, experimental studies, data acquisition, data analysis, statistical analysis, manuscript preparation, manuscript editing, and manuscript review. H.K. contributed to the literature search, clinical studies, experimental studies, data acquisition, data analysis, statistical analysis, manuscript preparation, manuscript editing, and manuscript review. H.S. contributed to the concept, design, definition of intellectual content, manuscript editing, manuscript supervision, and manuscript review. P.N. contributed to the concept, design, definition of intellectual content, manuscript editing, manuscript supervision, and manuscript review. M.K. contributed to the concept, design, definition of intellectual content, manuscript editing, manuscript supervision, and manuscript review. M.R.M. contributed to data analysis and statistical analysis.

## Funding

No funding was received for this manuscript.

## Disclosure

All authors agree to be accountable for the research presented.

## Conflicts of Interest

The authors declare no conflicts of interest.

## Data Availability

The data that support the findings of this study are available from the corresponding author upon reasonable request.
